# Using a Socioeconomic Position Index to Assess Disparities in Cancer Incidence and Mortality, Puerto Rico, 1995-2004

**Published:** 2011-12-15

**Authors:** Mariela Torres-Cintrón, Ana P. Ortiz, Karen J. Ortiz-Ortiz, Nayda R. Figueroa-Vallés, Javier Pérez-Irizarry, Taína De La Torre-Feliciano, Gwendolyn Díaz-Medina, Erick Suárez-Pérez

**Affiliations:** University of Puerto Rico Comprehensive Cancer Center; University of Puerto Rico Comprehensive Cancer Center, San Juan, Puerto Rico. Dr Ortiz is also affiliated with the Graduate School of Public Health, Medical Sciences Campus, University of Puerto Rico, San Juan, Puerto Rico; Puerto Rico Central Cancer Registry, Cancer Control and Population Sciences Program, University of Puerto Rico Comprehensive Cancer Center, San Juan, Puerto Rico; Puerto Rico Central Cancer Registry, Cancer Control and Population Sciences Program, University of Puerto Rico Comprehensive Cancer Center, San Juan, Puerto Rico; Puerto Rico Central Cancer Registry, Cancer Control and Population Sciences Program, University of Puerto Rico Comprehensive Cancer Center, San Juan, Puerto Rico; Puerto Rico Central Cancer Registry, Cancer Control and Population Sciences Program, University of Puerto Rico Comprehensive Cancer Center, San Juan, Puerto Rico; Natural Sciences Faculty, Río Piedras Campus, University of Puerto Rico, San Juan, Puerto Rico; Graduate School of Public Health, Medical Sciences Campus, University of Puerto Rico, San Juan, Puerto Rico

## Abstract

**Introduction:**

Evaluation of the extent of socioeconomic inequalities in cancer incidence and mortality is essential to generate hypotheses in population health research and provides evidence for population-based strategies for comprehensive cancer control. The objective of this study was to create an area-based socioeconomic position (SEP) index to assess possible socioeconomic disparities in incidence and mortality of selected cancers in Puerto Rico.

**Methods:**

Data for cancer incidence and mortality from 1995 to 2004 were obtained from the Puerto Rico Central Cancer Registry and the Puerto Rico Department of Health, and Puerto Rico socioeconomic data were obtained from the US Census 2000. We used principal component and factor analysis methods to construct the SEP index at the municipality level. We calculated age-adjusted incidence and mortality for each SEP area and used rate ratios to evaluate the differences by SEP.

**Results:**

Incidence and mortality of cancer in Puerto Rico varied by SEP area. In general, the incidence and mortality for cancers of the esophagus and stomach were higher for municipalities with the lowest SEP; in contrast, rates for breast, colorectal, kidney, pancreas, prostate, and thyroid were higher for areas with the highest SEP.

**Conclusion:**

These results highlight cancer disparities in Puerto Rico by SEP level that warrant further research.

## Introduction

Cancer is the second leading cause of death in Puerto Rico (1), a geographically diverse archipelago comprising 78 municipalities, with an estimated population of 3.9 million in 2007. Data from the Census 2000 indicate that socioeconomic disparities exist geographically across Puerto Rico, where municipalities differ by socioeconomic determinants such as proportion of residents living below the poverty level and with lower educational attainment. Socioeconomic disparities influence patterns of cancer morbidity and mortality (2) and may result in cancer disparities.

Cancer disparities are defined as adverse differences in cancer incidence, prevalence, death, survivorship, and burden of cancer or related health conditions among specific population groups (3). Socioeconomic position is a major cause of health disparities worldwide and is closely related to social class (4,5). Socioeconomic characteristics have long been studied in relation to health, disease, and mortality differences in the United States and other industrialized countries (6,7). Socioeconomic position is an aggregate concept with 2 main components: 1) material and social resources and assets such as access to and consumption of goods and services and 2) knowledge, such as occupational prestige, income, and educational level (4,5).

Evaluation of the extent of inequalities in cancer incidence and mortality is essential to generate hypotheses in population health research and provides evidence for population-based strategies for comprehensive cancer control. No consensus exists in the United States regarding which indicators should be used to measure socioeconomic inequalities and at which level of geography they should be measured and monitored (2,7). No measure exists for monitoring inequalities in health related to socioeconomic status in Puerto Rico. The objective of this study was to create an area-based socioeconomic position (SEP) index using the US Census 2000 for Puerto Rico, and to use this index to identify socioeconomic disparities in cancer incidence and mortality for selected cancers in Puerto Rico.

## Methods

We obtained cancer statistics from 1995 to 2004 from the Puerto Rico Central Cancer Registry (PRCCR) (8). Data for the incidence analysis included all cancers except squamous and basal cell carcinomas and in situ tumors of the uterine cervix. In 2003, a Centers for Disease Control and Prevention audit concluded that 95.3% of all cancer cases diagnosed or treated in hospital facilities in Puerto Rico were appropriately reported to the PRCCR, a result comparable to the US median of 95% (9). For specific cancer types, incident tumors were classified by primary site and histology, according to the *International Classification of Diseases for Oncology, Third Edition* (10). To be eligible for the analyses, cancer cases had to meet the following inclusion criteria: be incident cancer cases in patients who were residents of Puerto Rico at the time of diagnosis, have information on age and type of diagnostic confirmation, and have information on municipality of residence (96.1%). We obtained cancer mortality data from the Puerto Rico Department of Health (11) and used Puerto Rico socioeconomic data for each municipality from the 2000 US Census (12).

We used principal component (PC) and factor analysis methods to construct the SEP index by municipality level (13). Initially, we considered 14 indicators available in the US Census 2000 that describe the socioeconomic conditions in a community. We used the most correlated indicators for the PC analysis, based on the Pearson correlation index and Puerto Rico data. The estimated Pearson correlation index greater than 0.5 was the main operational criterion to define the most correlated indicators. The following 8 indicators were selected on the basis of this correlation assessment, their theoretical relevance, and prior empirical research (2,7): unemployment rate; median annual household income; percentage of the population living below the poverty level; percentage of the population aged 25 years or older with less than 12 years of education; percentage of occupied housing units without a car; percentage of the employed civilian population aged 16 years or older in management, professional, and related occupations (used to define white-collar occupations); percentage of occupied housing units without a telephone; and percentage of the population fluent in both English and Spanish (Appendix A). Using these 8 indicators, we performed the PC analysis to determine the number of potential components that would define an index or indexes of socioeconomic position; we standardized the indicator variables using the *z* score for each indicator. We reversed values for median household income, white-collar employment, and English language proficiency before computing the *z* score so that a higher score corresponded to a higher SEP score.

The PC analysis indicated that the first component showed 90.1% (*first eigenvalue*, *λ*_1_ = 5.25) of the total variance; therefore, only 1 factor was used to define the socioeconomic position index. Afterward, we performed a factor analysis (*X_i_* = *α*_1*i*_ *F*_1_ +  *e*_i_) to determine what percentage of the variance that 1 factor would explain of the total variance of each indicator; the squares of all factor loadings in this factor were above 31.4% (Appendix B).

To compute the SEP index, we multiplied each of the score coefficients of the first PC by the corresponding *z* score associated with each of the 8 socioeconomic indicators. The proportion of total variance explained by the first PC was 62%. To assess the internal consistency among the indicators that made up the index, we computed the Cronbach α using the 8 indicators used in the PC analysis; the result showed an α of 0.93, which indicates a high degree of internal consistency among the indicators that made up the index.

After we computed the socioeconomic index for each municipality, we defined 5 categories using the quintiles to set the scale boundaries, where SEP 1 represents the lowest socioeconomic level and the SEP 5 represents the highest socioeconomic level (Figure). Then, we calculated age-adjusted incidence and mortality for each SEP level and analyzed them in 2 periods (1995-1999 and 2000-2004), by sex and for overall cancer and selected cancer sites. We also calculated standardized rate ratios to evaluate the relative differences between the SEP levels. Significance was set at *P* < .05. We present data only for the comparison of the 2 extreme socioeconomic categories (SEP 1 vs SEP 5, reference group). We omit results for categories containing fewer than 15 cases. Rates were per 100,000 and age-adjusted to the Puerto Rico population according to the Census 2000.

**Figure.**
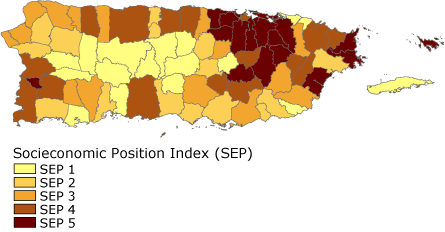
Municipalities by socioeconomic position (SEP) index, Puerto Rico, 2000. SEP 1 is the lowest socioeconomic position, and SEP 5 is the highest socioeconomic position. See Methods for calculations used to derive SEP scores.

## Results

### Socioeconomic position index

SEP 1 municipalities were concentrated in the central region of Puerto Rico, and the SEP 5 level municipalities were concentrated in and around the San Juan metropolitan area (Figure). The municipalities with the lowest SEP were Maricao, Guánica, and Las Marías, and the municipalities with the highest SEP were Guaynabo, Trujillo Alto, and Carolina.

### Cancer incidence

**Men**

During 2000-2004, the incidence of cancer for all sites among men was significantly lower in SEP 1 areas than in the SEP 5 areas (Table 1). Incidence of esophagus and stomach cancers were higher in SEP 1 than in SEP 5. During 2000-2004, incidence of the following cancers was lower in SEP 1 than in SEP 5: colorectal, kidney and renal pelvis, liver and intrahepatic bile duct, lung and bronchus, non-Hodgkin lymphoma, and prostate; a similar pattern was observed in the earlier time period (Table 1).

**Women**

During 2000-2004, the incidence of cancer for all sites among women was significantly lower in SEP 1 areas than in SEP 5 areas (Table 2). The incidence of esophagus and stomach cancers were higher in SEP 1 than in SEP 5 for the earlier period only. During 2000-2004, the incidence of the following cancers was lower in SEP 1 than in SEP 5: breast, colorectal, non-Hodgkin lymphoma, and thyroid; a similar pattern was observed in the earlier time period.

### Cancer mortality

**Men**

During 2000-2004, there was no difference in all-site cancer mortality among men in SEP 1 areas and those in SEP 5 areas (Table 3). Mortality due to esophagus and stomach cancers was higher in SEP 1 than in SEP 5. During 2000-2004, mortality was lower for the following cancers in SEP 1 than in SEP 5: colorectal, liver and intrahepatic bile duct, and pancreas. A similar pattern was observed in the earlier time period for colorectal and liver and intrahepatic bile duct cancers.

**Women**

During 2000-2004, the cancer mortality for all sites among women was significantly lower in SEP 1 areas than in SEP 5 areas (Table 4). During 2000-2004, mortality for breast cancer was lower in the SEP 1 than the SEP 5 areas; similar patterns were observed in the earlier time period.

## Discussion

This study creates for the first time a composite measure of area-based SEP for the analysis of socioeconomic disparities in cancer incidence and mortality in Puerto Rico. This is also the first study to develop area-based socioeconomic measures for monitoring population health in Puerto Rico, although several studies report the SEP of Puerto Ricans living in the continental United States (14,15). Our study shows SEP differences by municipalities and links those differences to differences in cancer incidence and mortality throughout Puerto Rico. These SEP differences could be a source of potential differences in lifestyle and environmental exposures throughout Puerto Rico, such as access to care (7), but we do not have data to support this explanation.

### Socioeconomic position index

Our study shows that more deprived municipalities are concentrated in the central region of the Island, and less deprived municipalities are concentrated in the San Juan metropolitan area. People living in the mountainous center of Puerto Rico may lack access to health services because of economic, cultural, environmental, social, and physical barriers (7,15-17). In Puerto Rico, as in the continental United States, one of the most important determinants of access to medical care is health insurance coverage (18). Puerto Rico has a population of 3.9 million people, and approximately 91.6% are insured by public or private health insurance. Approximately 8.4% of the population is uninsured, similar to estimates of the proportion of uninsured people in United States (19). Two health care systems coexist in Puerto Rico: the private system consisting of private health insurers and Medicare (parts A and B), and the public system (Reforma) that serves more than half of the population. Although Reforma is administered by a single government agency, different insurance companies serve the 8 health regions of Puerto Rico, creating a heterogeneous model of health services. The private sector is the largest provider of services in Puerto Rico, although the government is responsible for most high-risk cases because most tertiary care services are provided by the state through the facilities of the Río Piedras Medical Center. According to the Registry of Hospitals and Health Facilities for 2002-2004, Puerto Rico has 67 hospitals, 16.7% of which are public. The distribution of hospitals varies by health region, and most are concentrated in the San Juan metropolitan region (20), an area that has high SEP. Thus, differences in access to care throughout Puerto Rico may also explain some of the differences observed in the incidence and mortality of cancer by SEP area.

### Cancer incidence

We observed a positive socioeconomic gradient for overall cancer incidence, with higher cancer rates in SEP 5, and a difference of approximately 13% between high and low SEP levels for 2000-2004. This difference could be explained by the lack of access to and use of medical care in the SEP 1 areas. Most of the SEP 1 areas are in the central region of Puerto Rico, where fewer clinical facilities are available (16). Thus, the lower incidence of cancer in these regions could be partially explained by underdiagnosis.

Consistent with patterns in the incidence of all sites, incidence of the most common cancer types in Puerto Rico (breast, colorectal, and prostate) was higher in the SEP 5 areas, where most mammography facilities, urologists, and gastroenterologists are clustered (16,21). Given that these cancers are susceptible to screening and thus to potential overdiagnosis (21,22) in regions with better access to health care facilities, the proportion of cases underdiagnosed may be lower than in regions with poorer access to care (21,22). Future studies should explore whether the clustering of health care facilities in Puerto Rico influences disease detection in this population.

The other cancer types that showed higher incidence in SEP 5 areas were kidney, liver, lung in men, thyroid in women, and lymphoma. Differences in the occurrence of risk factors for these cancer types between the SEP levels could partially explain this pattern (23,24). Contrary to our data, according to the 2002 Behavioral Risk Factor Surveillance System (BRFSS) in Puerto Rico, the prevalence of smoking was higher among residents with lower incomes and lower levels of education, as in the continental United States. Nonetheless, income is only one of the variables included in our SEP index, and thus, these patterns may not be comparable. More research is necessary to evaluate whether in Puerto Rico SEP influences smoking behavior and other behavioral risk factors.

Higher rates of liver cancer among men in the SEP 5 areas may reflect the higher prevalence of alcohol consumption in this group. In a recent study in Puerto Rico, the prevalence of hepatitis C virus infection did not vary according to education and annual family income (25,26). In addition, the incidence of 1 cancer associated with human papillomavirus, penile cancer, was higher in the SEP 1, although this difference was significant only during 1 period. This finding is consistent with those of some studies that show that people who live in counties with higher poverty have higher rates of penile cancer (27).

### Cancer mortality

Overall cancer mortality did not differ greatly by SEP, but mortality for specific cancer sites did. In general, we found higher mortality for stomach cancer for both sexes, and for esophagus cancer in men, in SEP 1 than in SEP 5. This result is consistent with those of other studies that show that mortality for stomach and esophagus cancers is high in areas with low socioeconomic status (7). The principal infectious agent associated with stomach cancer morbidity and mortality worldwide is *Helicobacter pylori* (25,28). This infection is associated with socioeconomics factors such as low income and education level (25,28). For esophagus cancer, the higher prevalence of behavioral risk factors in the SEP 1, including tobacco use, consumption of hard liquor, and a deficient diet (29), could partially explain the higher mortality. For example, BRFSS data indicate that during 2004 in Puerto Rico, the prevalence of current smoking was higher and the consumption of fruits and vegetables was lower in people with lower income and lower educational attainment (19).

This study has several limitations. Puerto Rico cancer data do not permit analysis for smaller geographic areas, such as subcounty level, census tract, or block groups, because the address information is not available at this level for most of the cases. However, municipality-level data provide an appropriate socioeconomic and political context for the formulation and implementation of public health and social policies. Area-based socioeconomic variations in cancer incidence and mortality cannot be considered as proxies for socioeconomic differentials at the individual level. Such consideration may lead to the ecological fallacy (7). Whereas area socioeconomic patterns for several of the cancer outcomes are generally consistent with those at the individual level, the area-level effects we found may differ in magnitude from individual socioeconomic effects. These differences may be partly due to the compositional heterogeneity of the areas examined, particularly municipalities, which, unlike subcounty levels or census tracts, may contain substantial socioeconomic variability (2,7).

In conclusion, this study identifies disparities in cancer incidence and mortality that could be due to health care access and utilization rather than actual disease incidence; our findings warrant further investigation.

## Figures and Tables

**Table 1:** Age-Standardized Incidence^a^ for Different Cancer Sites Among Men, by Socioeconomic Position (SEP) Index,^b^ Puerto Rico, 1995-2004

Cancer Type	1995-1999			2000-2004		

	SEP 1^c^	SEP 5^d^	SRR (95% CI)^e^	SEP 1^c^	SEP 5^d^	SRR (95% CI)^e^
All sites	306.5	350.9	0.87 (0.83-0.91)	299.9	332.8	0.90 (0.86-0.94)
Brain and other nervous system	4.1	5.1	0.79 (0.52-1.18)	3.8	3.6	1.05 (0.68-1.56)
Colon and rectum	31.2	41.9	0.74 (0.64-0.85)	32.5	42.6	0.76 (0.66-0.87)
Esophagus	11.5	7.9	1.45 (1.11-1.87)	10.2	6.6	1.54 (1.18-1.99)
Kidney and renal pelvis	3.8	7.1	0.53 (0.34-0.80)	4.3	7.6	0.56 (0.38-0.81)
Larynx	8.3	8.8	0.94 (0.69-1.25)	7.8	6.3	1.25 (0.92-1.66)
Leukemia	6.8	8.6	0.79 (0.57-1.07)	6.7	7.3	0.91 (0.66-1.22)
Liver and intrahepatic bile duct	6.6	10.7	0.61 (0.44-0.83)	7.1	11.0	0.64 (0.47-0.86)
Lung and bronchus	25.1	27.5	0.91 (0.77-1.07)	19.4	24.1	0.80 (0.67-0.95)
Non-Hodgkin lymphoma	9.7	12.2	0.79 (0.60-1.02)	7.9	11.9	0.66 (0.49-0.86)
Oral cavity and pharynx	18.1	17.8	1.01 (0.83-1.23)	14.7	13.9	1.05 (0.85-1.29)
Pancreas	6.6	6.7	0.98 (0.69-1.35)	4.8	6.3	0.75 (0.51-1.06)
Penis	4.0	2.3	1.74 (1.09-2.71)	2.6	1.5	1.69 (0.96-2.87)
Prostate	101.3	128.7	0.78 (0.72-0.85)	114.4	128.8	0.88 (0.82-0.95)
Stomach	26.3	13.3	1.97 (1.64-2.35)	17.2	11.1	1.54 (1.26-1.89)
Thyroid	NC^f^	NC^f^	NC^f^	2.1	2.7	0.77 (0.43-1.30)
Urinary bladder	11.8	12.4	0.94 (0.73-1.20)	11.3	11.6	0.97 (0.76-1.23)

Abbreviations: SRR, standardized rate ratio; CI, confidence interval; NC, not calculated.

a Rates are per 100,000 and are age-adjusted to the Census 2000 population for Puerto Rico (12).

b See Methods for calculations used to derive SEP scores.

c SEP 1 is the lowest socioeconomic position.

d SEP 5 is the highest socioeconomic position and is the reference group.

e Defined as SEP 1 divided by SEP 5 according to the Tiwari method.

f Cell contains <15 cases in 1 of the categories.

**Table 2:** Age-Standardized Incidence^a^ for Different Cancer Sites Among Women, by Socioeconomic Position (SEP) Index,^b^ Puerto Rico, 1995-2004

Cancer Type	1995-1999			2000-2004		

	SEP 1^c^	SEP 5^d^	SRR (95% CI)^e^	SEP 1^c^	SEP 5^d^	SRR (95% CI)^e^
All sites	184.6	223.1	0.82 (0.78-0.87)	184.5	219.4	0.84 (0.79-0.88)
Brain and other nervous system	1.9	3.6	0.52 (0.28-0.88)	3.4	3.2	1.06 (0.69-1.59)
Breast	49.9	75.3	0.66 (0.59-0.73)	54.9	73.9	0.74 (0.67-0.81)
Cervix uteri	9.6	10.0	0.96 (0.74-1.23)	8.1	6.9	1.17 (0.89-1.52)
Colon and rectum	23.4	27.9	0.83 (0.71-0.98)	24.4	30.0	0.81 (0.70-0.94)
Corpus and uterus, NOS	10.0	12.9	0.77 (0.60-0.98)	14.5	14.1	1.02 (0.83-1.24)
Esophagus	3.3	1.9	1.76 (1.07-2.77)	2.3	1.4	1.71 (0.98-2.85)
Kidney and renal pelvis	2.3	3.1	0.74 (0.42-1.21)	2.3	3.3	0.70 (0.41-1.12)
Larynx	NC^f^	NC^f^	NC^f^	NC^f^	NC^f^	NC^f^
Leukemia	4.4	5.1	0.85 (0.57-1.22)	4.5	4.8	0.95 (0.65-1.33)
Liver and intrahepatic bile duct	4.6	3.6	1.27 (0.85-1.83)	3.7	3.7	0.98 (0.65-1.44)
Lung and bronchus	8.7	10.1	0.86 (0.65-1.12)	9.2	10.5	0.87 (0.68-1.11)
Non-Hodgkin lymphoma	6.6	8.6	0.76 (0.55-1.02)	5.5	8.3	0.65 (0.47-0.89)
Oral cavity and pharynx	3.7	3.8	0.96 (0.62-1.44)	3.3	3.8	0.86 (0.55-1.27)
Pancreas	4.7	5.4	0.86 (0.58-1.22)	3.2	4.5	0.71 (0.46-1.06)
Stomach	10.4	7.0	1.49 (1.14-1.91)	7.3	5.9	1.24 (0.93-1.64)
Thyroid	5.1	7.0	0.71 (0.50-0.99)	5.3	10.5	0.50 (0.36-0.67)
Urinary bladder	4.9	4.6	1.06 (0.73-1.51)	2.8	3.5	0.80 (0.50-1.22)

Abbreviations: SRR, standardized rate ratio; CI, confidence interval; NOS, not otherwise specified; NC, not calculated.

a Rates are per 100,000 and are age-adjusted to the Census 2000 population for Puerto Rico (12).

b See Methods for calculations used to derive SEP scores.

c SEP 1 is the lowest socioeconomic position.

d SEP 5 is the highest socioeconomic position and is the reference group.

e Defined as SEP 1 divided by SEP 5 according to the Tiwari method.

f Cell contains <15 cases in 1 of the categories.

**Table 3:** Age-Standardized Mortality^a^ for Different Cancer Sites Among Men, by Socioeconomic Position (SEP) Index,^b^ Puerto Rico, 1995-2004

Cancer Type	1995-1999			2000-2004		

	SEP 1^c^	SEP 5^d^	SRR (95% CI)^e^	SEP 1^c^	SEP 5^d^	SRR (95% CI)^e^
All sites	172.0	170.4	1.00 (0.94-1.07)	151.4	155.6	0.97 (0.91-1.03)
Brain and other nervous system	NC^f^	NC^f^	NC^f^	1.1	2.3	0.50 (0.22-1.00)
Colon and rectum	11.9	17.3	0.68 (0.53-0.86)	13.1	18.1	0.72 (0.58-0.89)
Esophagus	11.5	7.7	1.50 (1.15-1.94)	8.0	5.9	1.37 (1.01-1.83)
Kidney and renal pelvis	2.0	2.2	0.94 (0.49-1.67)	2.2	2.4	0.92 (0.51-1.56)
Larynx	4.5	4.5	1.01 (0.66-1.49)	3.4	3.1	1.10 (0.69-1.69)
Leukemia	5.9	5.1	1.15 (0.79-1.62)	5.0	5.5	0.91 (0.63-1.28)
Liver and intrahepatic bile duct	9.4	12.0	0.78 (0.59-1.01)	8.6	12.4	0.69 (0.52-0.89)
Lung and bronchus	24.3	25.1	0.96 (0.81-1.14)	18.2	23.5	0.77 (0.64-0.92)
Non-Hodgkin lymphoma	4.0	5.1	0.78 (0.50-1.16)	4.5	5.4	0.81 (0.55-1.17)
Oral cavity and pharynx	8.1	6.7	1.21 (0.88-1.62)	6.0	5.5	1.10 (0.78-1.52)
Pancreas	8.0	6.9	1.15 (0.84-1.55)	4.3	6.7	0.64 (0.43-0.92)
Penis	NC^f^	NC^f^	NC^f^	NC^f^	NC^f^	NC^f^
Prostate	30.5	37.7	0.80 (0.69-0.93)	35.1	31.1	1.12 (0.98-1.29)
Stomach	19.0	10.5	1.80 (1.46-2.22)	13.1	8.2	1.59 (1.25-2.00)
Thyroid	NC^f^	NC^f^	NC^f^	NC^f^	NC^f^	NC^f^
Urinary bladder	3.6	3.3	1.09 (0.67-1.72)	3.7	3.5	1.07 (0.68-1.63)

Abbreviations: SRR, standardized rate ratio; CI, confidence interval; NC, not calculated.

a Rates are per 100,000 and are age-adjusted to the Census 2000 population for Puerto Rico (12).

b See Methods for calculations used to derive SEP scores.

c SEP 1 is the lowest socioeconomic position.

d SEP 5 is the highest socioeconomic position and is the reference group.

e Defined as SEP 1 divided by SEP 5 according to the Tiwari method.

f Cell contains <15 cases in 1 of the categories.

**Table 4:** Age-Standardized Mortality^a^ for Different Cancer Sites Among Women, by Socioeconomic Position (SEP) Index,^b^ Puerto Rico, 1995-2004

Cancer Type	1995-1999			2000-2004		

	SEP 1^c^	SEP 5^d^	SRR (95% CI)^e^	SEP 1^c^	SEP 5^d^	SRR (95% CI)^e^
All sites	87.9	94.9	0.92 (0.85-1.00)	83.5	91.8	0.90 (0.83-0.98)
Brain and other nervous system	NC^f^	NC^f^	NC^f^	NC^f^	NC^f^	NC^f^
Breast	11.6	18.2	0.63 (0.50-0.79)	13.0	18.5	0.70 (0.57-0.85)
Cervix uteri	NC^f^	NC^f^	NC^f^	1.8	2.3	0.81 (0.44-1.37)
Colon and rectum	9.8	11.3	0.86 (0.67-1.10)	11.5	11.3	1.01 (0.81-1.26)
Corpus and uterus, NOS	3.1	3.4	0.90 (0.56-1.39)	4.2	3.2	1.31 (0.88-1.88)
Esophagus	2.9	2.0	1.43 (0.85-2.29)	1.8	1.0	1.76 (0.91-3.16)
Kidney and renal pelvis	NC^f^	NC^f^	NC^f^	NC^f^	NC^f^	NC^f^
Larynx	NC^f^	NC^f^	NC^f^	NC^f^	NC^f^	NC^f^
Leukemia	2.9	3.2	0.89 (0.54-1.40)	2.8	3.7	0.74 (0.46-1.14)
Liver and intrahepatic bile duct	6.7	5.0	1.33 (0.96-1.81)	4.5	5	0.88 (0.61-1.24)
Lung and bronchus	9.2	9.8	0.94 (0.71-1.21)	9.8	9.9	0.98 (0.77-1.24)
Non-Hodgkin lymphoma	2.3	3.7	0.62 (0.35-1.01)	2.2	3.2	0.68 (0.39-1.09)
Oral cavity and pharynx	NC^f^	NC^f^	NC^f^	NC^f^	NC^f^	NC^f^
Pancreas	4.6	5.2	0.87 (0.59-1.24)	3.5	4.4	0.79 (0.52-1.16)
Stomach	7.5	4.5	1.64 (1.20-2.22)	5.2	4.1	1.26 (0.89-1.75)
Thyroid	NC^f^	NC^f^	NC^f^	NC^f^	NC^f^	NC^f^
Urinary bladder	NC^f^	NC^f^	NC^f^	NC^f^	NC^f^	NC^f^

Abbreviations: SRR, standardized rate ratio; CI, confidence interval; NC, not calculated; NOS, not otherwise specified.

a Rates are per 100,000 and are age-adjusted to the Census 2000 population for Puerto Rico (12).

b See Methods for calculations used to derive SEP scores.

c SEP 1 is the lowest socioeconomic position.

d SEP 5 is the highest socioeconomic position and is the reference group.

e Defined as SEP 1 divided by SEP 5 according to the Tiwari method.

f Cell contains <15 cases in 1 of the categories.

## Appendices

### Appendix A. Correlations^a^ Among Socioeconomic Variables, 78 Municipalities, Puerto Rico, 2000

**Table:**

Variable^b^	Variable													

	A	B	C	D	E	F	G	H	I	J	K	L	M	N
A: Unemployment	1													
B: Median annual household income	−0.81	1												
C: Poverty	0.81	−0.93	1											
D: Expensive house	−0.40	0.49	−0.38	1										
E: >12th grade education	0.60	−0.84	0.86	−0.38	1									
F: Crowding	0.36	−0.40	0.52	−0.28	0.39	1								
G: No car	0.57	−0.61	0.57	−0.11	0.38	0.40	1							
H: Renter house	−0.01	−0.01	−0.06	0.16	−0.11	−0.21	0.29	1						
I: White collar	−0.52	0.65	−0.56	0.64	−0.59	−0.24	−0.22	0.35	1					
J: Single-parent household	0.01	0.12	−0.19	0.19	−0.41	−0.24	0.30	0.36	0.19	1				
K: No telephone	0.71	−0.78	0.79	−0.52	0.72	0.50	0.44	−0.23	−0.65	−0.26	1			
L: Plumbing facilities	0.46	−0.45	0.45	−0.26	0.41	0.15	0.26	−0.25	−0.44	0.01	0.51	1		
M: English proficiency	−0.55	0.66	−0.74	0.54	−0.60	−0.58	−0.27	0.22	0.42	0.26	−0.63	−0.30	1	
N: Working class	0.08	−0.12	0.11	−0.15	0.02	0.15	0.15	−0.09	−0.30	0.22	0.01	0.08	0.04	1

a Calculated by using the Pearson correlation index.

b All variables defined by the 2000 US Census for Puerto Rico (12).

### Appendix B. Factor Loadings and Principal Component (PC) Score Coefficients to Define the Area Socioeconomic Position Index Derived at Municipality Level, Puerto Rico, 2000

**Table:**

**Census 2000 Information**	Factor Loading	PC Score Coefficient
Unemployment rate (% civilian labor force aged ≥16 y unemployed)	0.83	0.36
Median family income^a^	0.97	0.41
Poverty (% population with 1999 income below poverty level)	0.97	0.41
% Population aged ≥25 y with <12 y education	0.85	0.37
% Employed civilian population aged ≥16 y in management, professional, and related occupations^a^	0.65	0.30
% Occupied housing units without telephone	0.85	0.37
% Population aged ≥5 y that speaks Spanish and speaks English very well^a^	0.71	0.32
% Occupied housing units with no vehicle available	0.56	0.26

a Values were reversed before the *z* score was computed so that a higher score corresponded to a higher socioeconomic position index score.
